# Tablets at the bedside - iPad-based visual field test used in the diagnosis of Intrasellar Haemangiopericytoma: a case report

**DOI:** 10.1186/s12886-017-0445-z

**Published:** 2017-04-24

**Authors:** Nisha Nesaratnam, Peter B. M. Thomas, Ramez Kirollos, Algis J. Vingrys, George Y. X. Kong, Keith R. Martin

**Affiliations:** 1 Department of Ophthalmology, Addenbrooke’s Hospital, Cambridge University Hospitals NHS Foundation Trust, Hills Road, Cambridge, CB2 0QQ UK; 2 Department of Neurosurgery, Addenbrooke’s Hospital, Cambridge University Hospitals NHS Foundation Trust, Hills Road, Cambridge, UK; 3 Department of Optometry & Vision Sciences Melbourne School of Health Sciences, University of Melbourne, Melbourne, VIC 3010 Australia

**Keywords:** Melbourne rapid field (MRF), Intrasellar haemangiopericytoma, Bitemporal hemianopia, Translational technology, Case report

## Abstract

**Background:**

In the assessment of a pituitary mass, objective visual field testing represents a valuable means of evaluating mass effect, and thus in deciding whether surgical management is warranted.

**Case presentation:**

In this vignette, we describe a 73 year-old lady who presented with a three-week history of frontal headache, and ‘blurriness’ in the left side of her vision, due to a WHO grade III anaplastic haemangiopericytoma compressing the optic chiasm. We report how timely investigations, including an iPad-based visual field test (Melbourne Rapid Field, (MRF)) conducted at the bedside aided swift and appropriate management of the patient.

**Conclusions:**

We envisage such a test having a role in assessing bed-bound patients in hospital where access to formal visual field testing is difficult, or indeed in rapid testing of visual fields at the bedside to screen for post-operative complications, such as haematoma.

## Background

History, examination and investigation of a patient with a suspected pituitary mass should aim not only to identify the cause of the mass, but also to ascertain if there is compression of adjacent structures, or clinical features of pituitary hormone abnormality. In any patient with signs of mass effect, such as a visual field defect or cranial nerve neuropathy, urgent MRI imaging and consideration of surgical decompression is warranted [1].

Formal assessment of visual fields using standard automated perimetry is routinely performed in patients with pituitary tumours to determine the degree of impairment caused by optic chiasm compression. Such assessment is necessary in planning the urgency of surgical intervention. However, formal standard automated perimetry cannot be performed in patients who are bed-bound, or in situations when visual field testing equipment is unavailable. Here we report a case in which a novel visual field test using a portable tablet device (Melbourne Rapid Field, (MRF)) was used to assess a patient with a pituitary tumour at the bedside.

## Case presentation

A 73 year-old presented to Addenbrooke’s Hospital in Cambridge with a three-week history of frontal headache and ‘blurriness’ in the left side of her vision. She had no nausea, vomiting, diplopia, facial pain or paraesthesia, and had no symptoms of pituitary hormone abnormality on admission. She had, however, presented 7 months prior with nausea and vomiting. On this previous admission, she was found to be hyponatraemic, with a reduced cortisol of 26 nmol/L and impaired cortisol response to synacthen (peak 119 nmol/L), and was commenced on oral hydrocortisone. FSH and LH were within normal limits and an MRI head showed no obvious mass lesion within the sella. Her past medical history included long-standing hypothyroidism, for which she was taking levothyroxine, iron-deficiency anaemia, and left sacroilitis.

At presentation, pertinent examination findings included a reduced visual acuity (6/18 in both eyes giving 6/12 with pinhole) and bitemporal hemianopia to confrontation. Further neurological examinations were unremarkable, and there were no other cranial nerve abnormalities. Urgent MRI head, pituitary hormone assays and Ophthalmology review for formal visual acuity and visual field testing were requested.

**Fig. 1:**
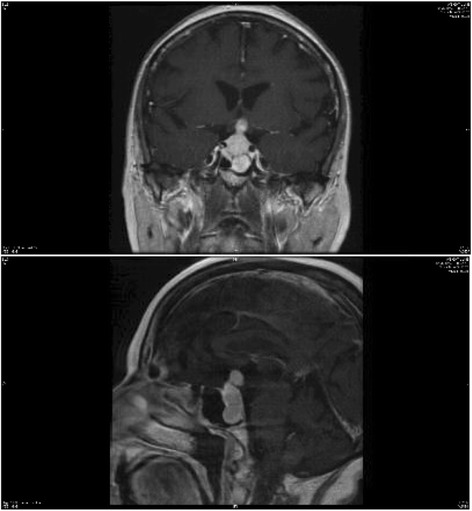
Sagittal and coronal MRI views, revealing a 34 × 12 × 15 mm intrasellar mass, extending into the sphenoid and cavernous sinuses

MRI imaging revealed an intrasellar mass with contrast enhancement (Fig. 1), which showed significant enlargement since the previous MRI head carried out 7 months earlier. Pre- and post-contrast images through the pituitary fossa showed the mass extending superiorly into the suprasellar region, where it appeared to compress the optic chiasm, and inferiorly into the sphenoid sinus. It had a lobular margin, and measured 34×12×15mm, with homogeneous enhancement post-contrast. It appeared to extend laterally into the cavernous sinus to lie inseparable from the carotid arteries. No pituitary tissue could be seen separately from the mass.

Ophthalmology review took place out-of-hours, when formal visual field testing was unavailable. Fundoscopy revealed healthy appearances of the optic disc. Visual acuity was 6/18 in both eyes. Visual field testing was performed on an iPad-based tangent perimeter, (Melbourne Rapid Field (MRF)), which performs fast thresholding at various locations within 30° of fixation, and has been validated in a patients with glaucoma [2]. With the iPad tablet screen, measuring 195 × 150 mm, viewed 33 cm away from the patient, the patient is instructed to fixate upon a target, and tap their finger on the screen or keyboard when they see a stimulus.

**Fig. 2:**
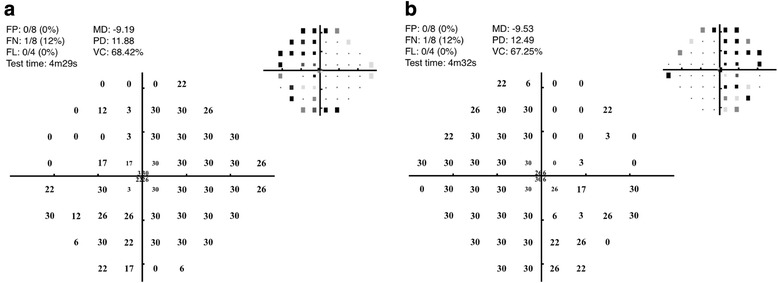
MRF result for left (**a**) and right (**b**) eyes, confirming a dense superior bitemporal field loss, with early inferior bitemporal involvement. Decibel threshold values are shown, with mean deviation (MD) and pattern deviation (PD) calculated therefrom. Insert at top right is a greyscale of depth of defect

The MRF test was performed at the bedside of the patient using an iPad tablet (iPad version 3, Apple, Cupertino USA) attached to a keyboard, and an eye occluder. Reliability tests including fixation loss, false positive and false negative rates were conducted throughout the test and the patient’s performance was within reliable limits. The test lasted 4.5 min for each eye, and confirmed a dense superior bitemporal field loss with early involvement of the inferior bitemporal fields (Fig. 2). The visual field loss involved both foveal visual fields and likely accounted for the drop in visual acuity.

**Fig. 3:**
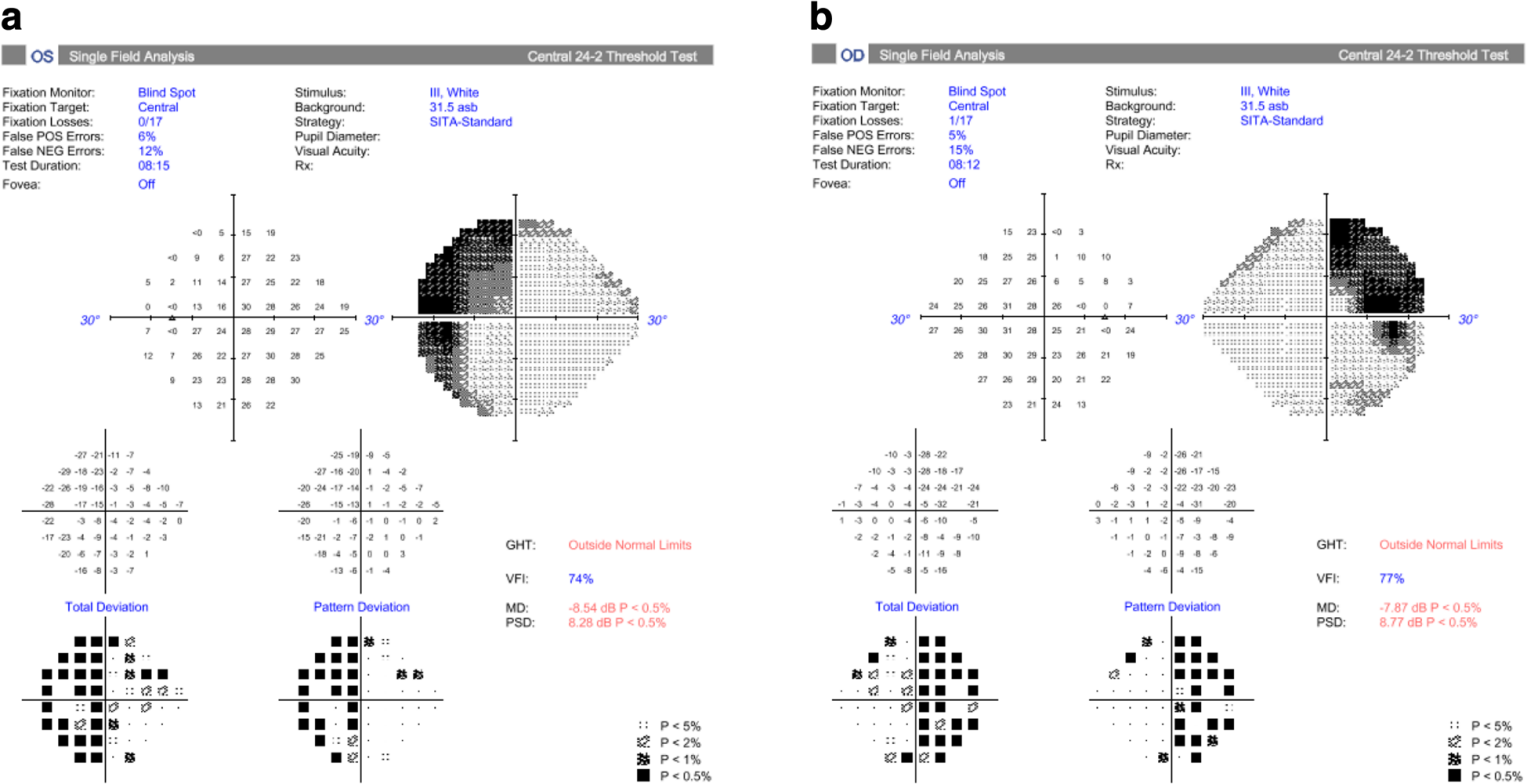
Humphrey 24–2 SITA-Standard visual field test result for patient’s left (**a**) and right (**b**) eyes, confirming a dense superior bitemporal field loss, conduced two days after MRF tests

**Fig. 4:**
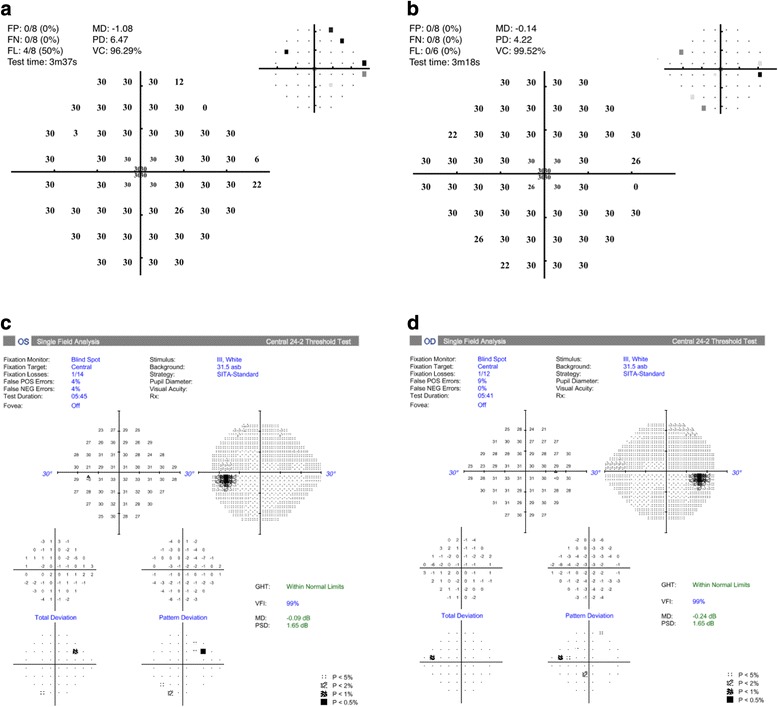
Melbourne Rapid Field (left - **a**, right - **b**) and Humphrey 24–2 SITA-Standard visual field test result (left - **c**, right - **d**), both confirmimg resolution of visual field defect two weeks post-operatively

With MRI imaging and objective visual field measurement confirming compression of the optic chiasm, neurosurgical intervention was planned, and the patient started on dexamethasone. Formal Humphrey 24–2 visual field assessment was performed 2 days later (Figs. 3 and 4) and confirmed a superior bitemporal visual field loss. Of note, global indices such as mean deviation and pattern deviation were comparable between MRF and Humphrey visual field tests. Mean deviation for the right eye was −9.53 dB (decibels) and −7.87 dB for MRF and Humphrey visual field respectively, and left eye was −9.19 dB and −8.54 dB respectively. Test time for Humphrey 24–2 field was longer, taking approximately 8 min per eye.

Two days later, the patient underwent an endonasal endoscopic resection of the pituitary mass. Histology revealed a WHO grade III anaplastic haemangiopericytoma, with a high proliferative grade (MIB-1 23%), pleomorphism and atypical cells. Given these pathological findings, and the high risk of recurrence and metastasis, FDG PET and bone scans were carried out. These revealed areas of uptake in the humeri, right sacral ala and small lymph nodes in the neck. Following a multi-disciplinary endocrine team discussion, the patient was started on a course of radiotherapy (54–60 Gy over 6 weeks), with a plan for early post-operative MRI head.

Two weeks post-operatively, the patient’s visual acuity had returned to its baseline of 6/9 in the right eye, and 6/6 in left eye (6/4 and 6/5 with pinhole). Repeat visual field testing using MRF and Humphrey 24–2 assessment was performed, and both showed marked improvement in the visual fields consistent with the improved acuity.

## Discussion and Conclusions

Tumours within the pituitary gland are common, with adenomas most prevalent and accounting for approximately 17% of lesions [3]. Intracranial haemangiopericytomas, first characterised by Stout and Murray in1942 [4], are rare vascular tumours, originating from contractile pericytes that form the walls of meningothelial capillaries. They are highly aggressive neoplasms, with a high incidence of recurrence and metastasis [5]. Our case describes the first reported use of the Melbourne Rapid Field test in clinical practice to assess visual fields in a patient with a pituitary mass. In this case, a quick iPad-based test demonstrated a bitemporal field defect at the bedside, and allowed appropriate planning for surgical intervention. The test also was able to confirm the resolution of bitemporal field defect following surgery, taking 3.5 mins to do so.

This test may have a role in assessing bed-bound patients in hospital where access to formal visual field testing is difficult, or indeed in rapid testing of visual fields at the bedside to screen for post-operative complications, such as haematoma. Use of a keyboard as in this case, which the patient can tap when they see a stimulus, limits the visual dexterity needed to perform the test, and avoids changes in the alignment and distance of the iPad relative to the eye.

We envisage the test also being utilised in settings where formal visual field testing is not readily available, such as rural areas in developing countries [6], or in the home setting, where visual fields could be monitored in patients with slow growing pituitary adenomas. Such a setup may improve the overall experience of regular visual field testing, and alleviate the current difficulties associated with formal testing currently reported by patients [7].

## Abbreviations

FDG PET: Fludeoxyglucose positron emission tomography
FSH: Follicle-stimulating hormone
LH: Luteinising hormone
MRF: Melbourne rapid field
MRI: Magnetic resonance imaging
WHO: World Health Organisation
